# An approach for particle sinking velocity measurements in the 3–400 μm size range and considerations on the effect of temperature on sinking rates

**DOI:** 10.1007/s00227-012-1945-2

**Published:** 2012-05-22

**Authors:** Lennart Thomas Bach, Ulf Riebesell, Scarlett Sett, Sarah Febiri, Paul Rzepka, Kai Georg Schulz

**Affiliations:** Helmholtz-Zentrum für Ozeanforschung (GEOMAR), Düsternbrooker Weg 20, 24105 Kiel, Germany

## Abstract

The flux of organic particles below the mixed layer is one major pathway of carbon from the surface into the deep ocean. The magnitude of this export flux depends on two major processes—remineralization rates and sinking velocities. Here, we present an efficient method to measure sinking velocities of particles in the size range from approximately 3–400 μm by means of video microscopy (FlowCAM^®^). The method allows rapid measurement and automated analysis of mixed samples and was tested with polystyrene beads, different phytoplankton species, and sediment trap material. Sinking velocities of polystyrene beads were close to theoretical values calculated from Stokes’ Law. Sinking velocities of the investigated phytoplankton species were in reasonable agreement with published literature values and sinking velocities of material collected in sediment trap increased with particle size. Temperature had a strong effect on sinking velocities due to its influence on seawater viscosity and density. An increase in 9 °C led to a measured increase in sinking velocities of ~40 %. According to this temperature effect, an average temperature increase in 2 °C as projected for the sea surface by the end of this century could increase sinking velocities by about 6 % which might have feedbacks on carbon export into the deep ocean.

## Introduction

Sinking velocities of marine particles are generally determined by two major properties—size and excess density over seawater. Most organic particles within the oceans are relatively small (<100 μm) and their individual contribution to the downward flux of organic matter is therefore minor (Clegg and Whitfield 1990). However, in case small particles aggregate to form larger ones, they might eventually sink fast enough to end up below winter mixed layer depths without severe remineralization. Aggregation is mediated either by zooplankton that graze on small particles and enclose them in larger fecal pellets, or by highly adhesive organic materials (e.g., Pilskaln and Honjo 1987; Passow et al. 1994). The excess density of particles is primarily determined by the amount of inorganic ballast material incorporated in them. Ballast materials can be either lithogenic (e.g., dust) or biogenic (opal and calcium carbonate).

It has been hypothesized that organic carbon export into the deep ocean is tightly coupled to these ballasting materials (Armstrong et al. 2002; Francois et al. 2002; Klaas and Archer 2002; but see also Passow 2004), since particles associated with them sink considerably faster than those consisting of organic matter alone (e.g., Honjo 1976; Engel et al. 2009; Iversen and Ploug 2010). Hence, a change in the ballast loading of particles may feedback on organic carbon export and consequently on atmospheric carbon dioxide levels (Riebesell et al. 2009). In this respect, sinking velocity measurements are a key parameter in estimating organic carbon export (e.g., Feinberg and Dam 1998; Ploug et al. 2008; Fischer and Karakas 2009).

Here, we introduce an efficient method to determine sinking velocities by means of video microscopy. This approach is easy to set up, can be applied to a wide particle size range, and allows precise measurement and analysis of high numbers of samples per day. Furthermore, particles within a mixed sample can be distinguished automatically during analysis so that only sinking velocities of particles fulfilling defined criteria are evaluated. It, therefore, offers new possibilities to determine sinking velocities of individual particle classes with high temporal resolution and without much effort over the course of long-lasting laboratory and field experiments.

## Materials and methods

### Sample preparation

Four different kinds of particles were prepared for sinking velocity measurements. These are small polystyrene beads, large polystyrene beads, monospecific phytoplankton cultures, and sediment trap material.

Small polystyrene beads (10 μm in diameter, Beckman) were delivered in an aqueous solution containing surfactants and preservatives. To remove this solution, they were rinsed on a polycarbonate filter (0.8-μm pore size) with 100 mL of filtered seawater. The beads that remained on the filter were resuspended in ~2 mL of filtered seawater (salinity = 35) and transferred using a pipette into the glass cuvette where sinking velocities were determined in. The larger beads (75–400 μm in diameter, Polysciences) were delivered as dry powder so that it was not necessary to rinse them. They were added directly to ~10 mL of filtered seawater (salinity = 33.5). In order to load the glass cuvette with the large beads, a 30-cm-long plastic pipe (inner diameter 6 mm) was connected to the top of the glass cuvette with a silicon tube. A pipetting ball was attached to the other side of the plastic pipe to suck the filtered seawater containing the large beads into the glass cuvette. Plastic pipe and pipetting ball remained on top of the glass cuvette during measurements and were stabilized with cable ties.

Monospecific phytoplankton cultures were grown in temperature and light-controlled conditions. The growth status and specific culture conditions of each species are given in Table 1. Approximately 2 mL of growth medium, containing the cells, was taken directly from the culture bottles and transferred into the glass cuvette which was used for measurements. In case cell densities of phytoplankton cultures were too low to get meaningful measurements, 60 ml of culture medium was concentrated by gravity on a 0.8-μm polycarbonate filter until approximately 1 mL was left. This concentrated cell suspension was then gently transferred using a pipette into the glass cuvette. Cell cultures concentrated this way are indicated in Table 1.

**Table 1:** Measured sinking velocities (corrected for wall effects with Eq. 3) of various phytoplankton species at individual culture conditions (temperature *T* (°C), salinity *S*, light (μmol m^−2^ s^−1^))

Species (strain)	Sinking velocity (m day^−1^)	ESD (μm)	*ρ*_particle_ (kg m^−3^)	*Re* (10^−5^)	*N*	Culture conditions
*Crocosphaera watsonii* (WH8501)	0.071 (±0.02)	3.77 (±0.61)	1,143.6 (±45.8)	0.29 (±0.12)	26	Nutrient replete; *T* = 28; *S* = 35; light = 150
*Dictyocha* spec. (isolated at N54° 19′ 48″ E10° 7′ 48″ in March 2011)	0.479 (±0.13)	11.38 (±1.3)	1,088.4 (±19.1)	6.33 (±2.38)	30	Nutrient replete; *T* = 15; *S* = 13.8; light = 150 Without visible silicate skeleton
*Rhodomonas* spec. (unknown)	0.25 (±0.06)	8.72 (±1.27)			50	Nutrient replete; *T* = 15; *S* = 15; light = 150 Only non-motile cells were investigated
*Emiliania huxleyi* (B92/11)	0.378 (±0.12)	6.41 (±1.12)	1,233.3 (±46.2)	2.84 (±1.33)	69	Nutrient replete; *T* = 15 °C; *S* = 35; light = 180
^a^ *Emiliania huxleyi* (B92/11)	0.301 (±0.11)	7.12 (±1.26)	1,160.5 (±41.1)	2.44 (±1.09)	22	3 days of phosphorus limitation; *T* = 15; *S* = 35; light = 150
*Emiliania huxleyi* without CaCO_3_ covering (B92/11)	0.034 (±0.01)	3.62 (±0.83)	1,090.6 (±30.5)	0.14 (±0.07)	31	Nutrient replete but grown at low dissolved inorganic carbon(~500 μmol kg^−1^) and low pH (~7.2) so that cells were not able to produce CaCO_3_. *T* = 15; *S* = 35; light = 150
^a^ *Gephyrocapsa oceanica* (RCC1303)	0.534 (±0.12)	8.04 (±0.68)	1,216 (±47.6)	4.75 (±1.25)	58	Nutrient replete; *T* = 20; *S* = 35; light = 150
*Gephyrocapsa oceanica* without CaCO_3_ covering (RCC1303)	0.053 (±0.12)	5.39 (±1.22)	1,071.5 (±27.5)	0.33 (±0.21)	49	Nutrient replete but grown at low dissolved inorganic carbon(~500 μmol kg^−1^) and low pH (~7.2) so that cells were not able to produce CaCO_3_. *T* = 15; *S* = 35; light = 150
*Calcidiscus leptoporus* (isolated at N38° 39′ 22″ W27° 14′ 08″ in April 2010)	4.312 (±1.31)	19.55 (±2.23)	1,281.5 (±63.3)	94.77 (±3.52)	110	Nutrient replete; *T* = 16; *S* = 35; light = 160
*Calcidiscus leptoporus* coccoliths (see above)	0.737 (±0.31)	6.73 (±1.25)			162	Nutrient replete; *T* = 16; *S* = 35; light = 160
*Thalassiosira oceanica* (CCMP1005)	0.202 (±0.15)	7.92 (±1.86)			17	Nutrient replete; *T* = 24.6; *S* = 35; light = 65
*Thalassiosira pseudonana* (CCMP1012)	0.13 (±0.11)	5.22 (±1.71)			16	Nutrient replete; *T* = 17; *S* = 35; light = 50
*Thalassiosira weissflogii* (CCMP1052)	0.068 (±0.05)	14.38 (±2.66)			22	Nutrient replete; *T* = 17; *S* = 35; light = 50
*Phaeodactylum tricornutum* (unknown)	0.065 (±0.04)	5.40 (±1.63)			60	Nutrient replete; *T* = 17; *S* = 35; light = 50

ESD is the equivalent spherical diameter, *Re* is the Reynolds number (dimensionless), and *N* displays the number of measured particles. All values are reported as means of all analyzed particles and corresponding standard deviations. pH is given on free scale. Cell densities that were increased by filtration as described in “Materials and methods” are marked with an uppercase a. No results are available for *ρ*_particle_ and *Re* in case particles are not spherical

Sediment trap material was obtained in spring 2011 during the SOPRAN mesocosm CO_2_ enrichment study off the coast of Bergen (Norway; 60° 15′ 36″ N 5° 12′ 0″ E). The material was collected for 24 h in the sediment trap and consisted to a large proportion of complete or fragmented fecal pellets produced by copepods (*Calanus finmarchicus*, *Calanus helgolandicus*, and *Temora* spec.) and appendicularians (probably *Oikopleura dioica*). The material was partitioned using a 300-μm sieve to remove large aggregates and gelatinous material which would have otherwise clogged the glass cuvette in which particle sinking velocity was determined. The particles passing through the sieve were collected in a petri dish and diluted with filtered seawater of known salinity (33.5). This sample was transferred into the glass cuvette in the same way as described for large polystyrene beads.

### Measurement of sinking velocities

**Fig. 1:**
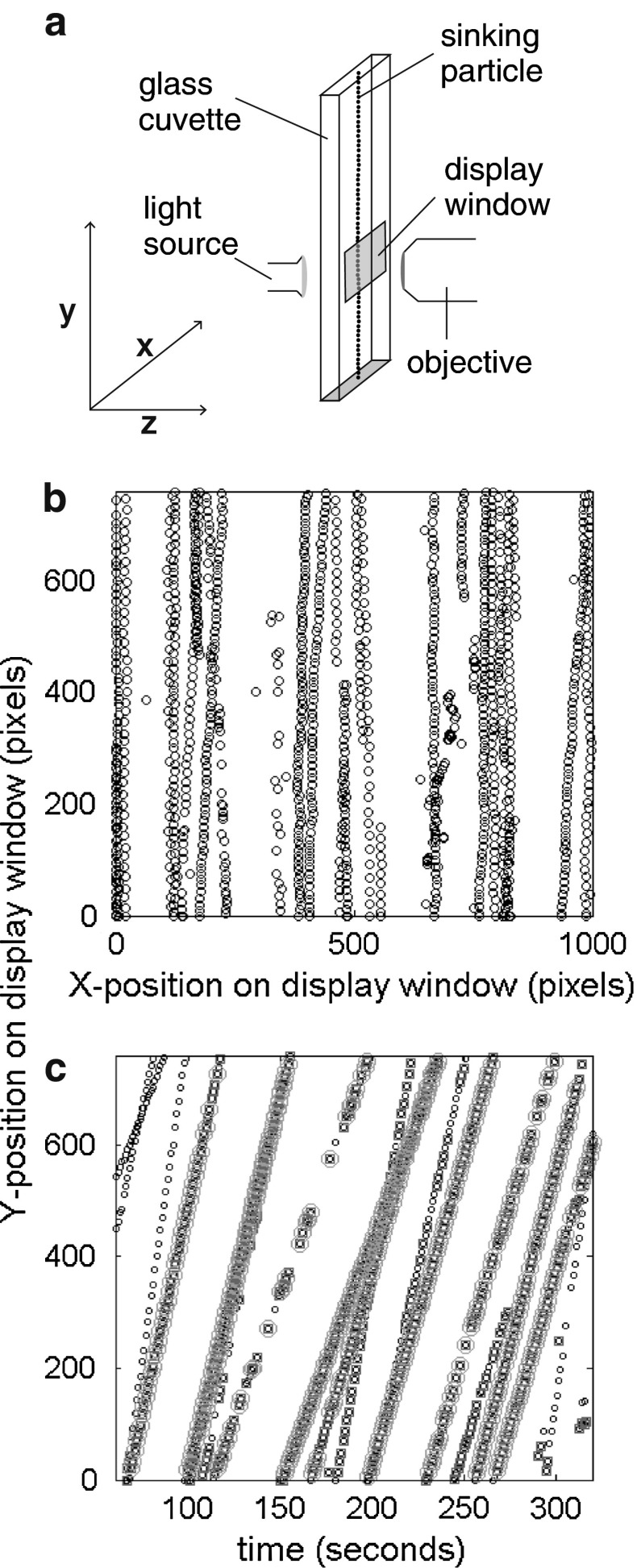
Evaluation of measured sinking velocities. **a** Sketch of the optical setup during a measurement. The *dotted line* denotes a particle that is sinking through the glass cuvette and photographed when passing the display window. **b** X- and Y-position of all particles photographed by the camera during the measurement. The *dotted lines* denote individual particles (in this case *Rhodomonas* spec.) falling vertically through the display window. Note irregularities in particle shape can cause small sidewards movements because particles start to glide to some extent. **c** Y-position of all measured particles on the display window plotted against the exact time the particles were photographed. The slopes of the *dotted lines* represent individual sinking velocities. The faster a particle is sinking, the steeper the slope. *Red squares* on *dots mark* those ones which were detected by the MATLAB analysis script as similar particle within a line. *Green circles* around marked *lines* are those particles which were finally evaluated since only the fits through these lines had *R*² ≥ 0.995

Sinking velocities were measured using the video microscope FlowCAM^®^ (Fluid Imaging), originally designed to characterize and count natural samples of plankton communities. For characterizations of plankton communities, the sample is pumped through a glass cuvette which is placed in front of a microscope camera so that particles inside the sample can be photographed and categorized by a software tool once they pass through the cuvette. For sinking velocity measurements, the pump was removed and the cuvette was closed airtight at the bottom and put vertically into the FlowCAM^®^ so that the particles inside the cuvette sink by gravity (Fig. 1a). Since the microscope camera was fixed, sinking particles could be photographed when they were sinking downward through the window monitored by the camera. The imaging system of the FlowCAM^®^ is composed of a CCD camera (Sony) with a resolution of 1,024 *×* 768 pixels (length × width) fitted with a microscope lens (Olympus). The field of view of the window monitored by the camera depended on the magnification of the microscope lens and ranged from ~8.5 mm^2^ (20 times magnification) to ~0.07 mm^2^ (200 times magnification). The camera was focused to the middle of the *x*- and *z*-plane of the cuvette (Fig. 1a). Particles that were photographed although not being entirely focused were discarded in a following step by evaluation script (see below). The FlowCAM^®^ is able to take up to 12 pictures per second. Faster sinking particles required higher frame rates to get enough pictures of individual particles because sinking particles should be photographed several times between entering and leaving the window monitored by the camera. The duration of a measurement depends on the average sinking velocity of the measured particles and on the particle density. It can be as short as ~3 min for dense samples with quickly sinking particles but can also last hours in case particle densities are low and they sink slowly. Small polystyrene beads and phytoplankton cells were measured for at least 30 min at 19 °C in a small glass cuvette (compare Table 2 for dimensions). Larger polystyrene beads and sediment trap material were measured in a large glass cuvette (Table 2) for at least 3 min at 10 and 19 °C, and 22 min at 10 °C, respectively. The magnification of the microscope lens had to be adjusted depending on the size of the measured particles. The large polystyrene beads and the sediment trap material were photographed with a ~20 times magnification, while the small polystyrene beads and the phytoplankton cells were ~200 times magnified. In practice, particles should not be smaller than 3 μm because it becomes difficult to distinguish cells from cell detritus in this size range. The upper size limit is not primarily set by the optics of the microscope but rather by the size of the glass cuvettes (see section on "Wall effects").

**Table 2:** Dimensions of glass cuvettes used for sinking velocity measurements

Glass cuvette	Length (mm)	Width (mm)	Depth (μm)
1	35	2.45	100
2	43	3.6	300
3	43	7	600
4	45	10	1,000
5	33.6	10	3,000

Small beads and phytoplankton sinking velocities were determined in cuvette 2, while large beads and sediment trap material sinking velocities were measured in cuvette 5

### Convection during measurements

Convection can result from temperature gradients within the glass cuvette and influence sinking velocities of particles. Temperature control is, therefore, essential and was closely monitored during all measurements. Furthermore, glass cuvettes were constantly ventilated with air to avoid the formation of local temperature gradients in the vicinity of heat sources. Measurements in glass cuvettes (larger polystyrene beads and sediment trap material; Table 2) were performed in either temperature-controlled rooms (variation ±0.3 °C) or light chambers for phytoplankton culturing (Rubarth Apparate GmbH, variation ±0.04 °C). Measurements in glass cuvettes <300 μm (small beads and phytoplankton cells; Table 2) were performed in temperature-controlled rooms (variation ±1 °C).

Evaporation at the air–water interface at the top of the glass cuvette is another potential source of convection as the water cools and becomes more saline, thereby inducing downwelling and upwelling. This can be minimized by sealing the top of the sedimentation column with a lid (Ploug et al. 2010). The 3,000-μm large glass cuvettes used for the measurements of large polystyrene beads and sediment trap material (Table 2) were sealed airtight by the pipetting ball used to transfer the sample into the sedimentation column (see section on “Sample preparation”). Glass cuvettes <3,000 μm (Table 2) were not entirely closed but a thin and ~5-cm-long silicon tube connected to the top of the cuvettes reduced evaporation since it minimized the gas exchange between the atmosphere and the air above the air–water interface.

The presence of convection cells was investigated by testing whether polystyrene beads of similar size and density sink constantly downward at every position inside the cuvette. We tested 100-, 300-, 600-, 1,000-, and 3,000-μm glass cuvettes (Table 2). Convection was usually present in glass cuvettes larger 300 μm at non-temperature-controlled conditions but was absent in all cuvettes when temperature was controlled. Convection was not detected in 100- and 300-μm glass cuvettes even in rooms with relatively large temperature variations of approximately ±1 °C. This might be due to adhesion effects in these comparatively thin and capillary-like glass cuvettes which prevent the formation of convection cells.

### Evaluation of sinking velocities

Each particle photographed by the FlowCAM^®^ was automatically characterized by 45 properties with the software “visual spreadsheet” (Fluid Imaging). The most important properties for the evaluation of sinking velocities were as follows: First, the horizontal and vertical position of the particles on the display window (X- and Y-coordinates) and the corresponding time the particle was photographed at that position. Second, several shape properties of the particle such as for example length, width, edge gradient, and equivalent spherical diameter (ESD). We developed a script using MATLAB (MathWorks^®^) which made use of these properties to automatically calculate sinking velocities together with mean particle length, width, and ESD of each particle. Furthermore, the script allowed definition of certain criteria so that only particles fulfilling these criteria were considered in the automatic evaluation. This ability was important (i) to discard particles that were not focused (by defining a minimum edge gradient of 0.8), and (ii) to distinguish, for example, fecal pellets and fecal pellet fragments within the sediment trap material from all other particles (by defining a minimum length of 150 μm and a maximum width to length ratio of 0.38). Note that these length values were determined visually before the evaluation with the MATLAB script. The basic functioning of the script shall be outlined in the following.

During measurement, particles are always sinking vertically through the area photographed by the camera (Fig. 1a) so that they change their position on the Y-coordinate in consecutive pictures but (if at all) only marginally on the X-coordinate. Since a sinking particle is photographed and characterized several times when sinking through the area monitored by the camera, its X- and Y-coordinates appear as vertical lines of dots when all coordinates are plotted into the same figure (Fig. 1b). Plotting the Y-position of every particle to the corresponding point in time instead of the corresponding X-position shows that sinking particles appear as separate lines of dots with certain slopes (Fig. 1c). The slopes of these lines depict the change of the vertical position of the particle per unit time and therefore their sinking velocity. The MATLAB script checks that all the dots within such line belong to the same particle by comparing several of their properties to each other (e.g., length, width, and ESD). Dots with inconsistent properties are removed from the line, while those with consistent properties are defined as one sinking particle. In the following step, the script fits a linear regression including only the dots with consistent properties. This regression is rejected from further evaluation by the script if the fit is based on less then 8 data points or if the coefficient of determination (*R* ^2^) is below 0.995. This second procedure erases irregular lines which accidentally pass the first sorting. In case a line of dots pass the two sorting procedures, the script stores the slopes of the regression lines as sinking velocity of the particles and calculates their average lengths, widths, and ESDs from the mean of all regression points. Furthermore, we implemented equations to calculate the Reynolds number of a sinking particle and its density.

The density of spherical particles (*ρ*_particle_) is calculated from Stokes’ law

1 $$ S_{\text{v}} = \frac{2}{9} \times g \times r^{2} \times \frac{{\rho_{\text{particle}} - \rho_{\text{seawater}} }}{{\eta_{\text{seawater}} }} $$

where *S*_v_ is the sinking velocity of a particle corrected for wall effects (see section on “Wall effects”), *η*_seawater_ is the dynamic viscosity of seawater (termed viscosity in the following for simplicity), g the Earth’s gravitational acceleration (9.81 m s^−2^), r the radius of the sphere, and *ρ*_seawater_ the density of seawater. Values for *η*_seawater_ and *ρ*_seawater_ are calculated from measured salinity and temperature according to Sharqawy et al. (2010). Note that particle densities are only calculated this way if the sinking particle was spherical, since other shapes change the drag and cause deviations of sinking velocities from Stokes’ Law (McNown and Malaika 1950).

The Reynolds number of sinking particles (*Re*) is calculated according to

2 $$ Re = \frac{{2 \times \rho_{\text{seawater}} \times S_{\text{v}} \times r}}{{\eta_{\text{seawater}} }} $$

*where Re* is an important measure to determine whether Stokes’ Law is applied to a sinking particle. Particle sinking velocities start to deviate from sinking velocities calculated with Stokes’ Law when *Re* > ~0.1–0.5 (McNown and Malaika 1950).

The evaluation script can be supplied by the corresponding author.

### Quantification of wall effects

**Fig. 2:**
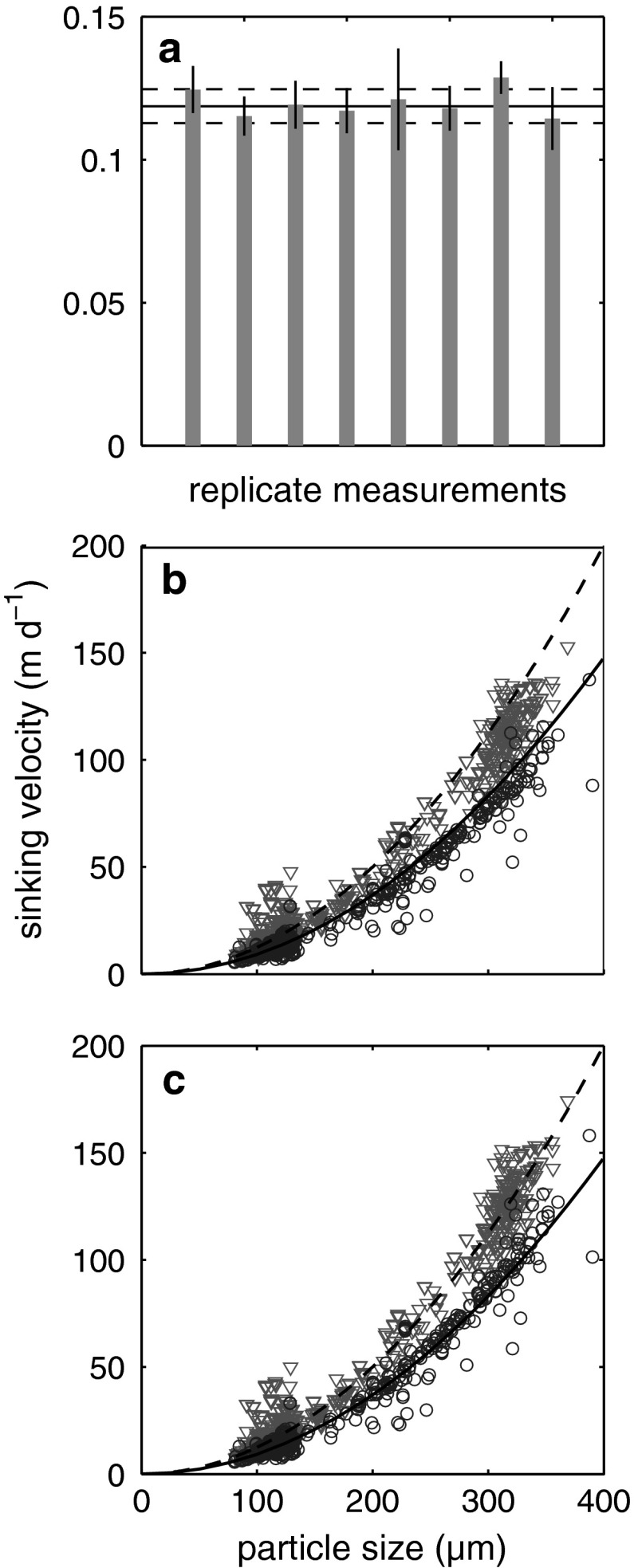
Sinking velocities of polystyrene beads. **a** Comparison of repeated sinking velocity measurements of polystyrene beads of known diameter (10 ± 0.09 μm) and density (1,053 kg m^−3^ ±0.3, Giddings and Ho (1995)) with the theoretical value calculated according to Stokes’ Law (Eq. 1). *Gray bars* denote the mean of on average 23 beads (±standard deviation). The *horizontal solid black line* depicts the theoretical sinking velocity calculated from Stokes’ Law, while the *dashed black lines* illustrate its upper and lower limits. These limits were calculated from an error propagation with uncertainties in size and *ρ*_particle_ given above, and an uncertainty of 2.6 10^−5^ kg m^−1^ s^−1^ in *η*_seawater_ and 0.3 10^−3^ kg m^−3^ in *ρ*_seawater_. The uncertainties in *η*_seawater_ and *ρ*_seawater_ are caused by an estimated uncertainty of 1 °C in the temperature-controlled room. **b** Measured sinking velocities in relation to size. *Red triangles* and *blue dots* are beads sinking in seawater of 19 and 10 °C, respectively. The *two lines* denote theoretical sinking velocities according to Stokes’ Law (*dashed line* for 19 °C, *straight line* for 10 °C). **c** Same data as in (**b**) but corrected for wall effects (Eq. 3)

The sinking velocity of particles decreases when sinking in close proximity to a wall (Happel and Brenner 1991). At Reynolds numbers below 0.5, such wall effects only depend on the distance of the particle to the wall (Uhlherr and Chhabra 1995). In this study, sinking velocities were measured in glass cuvettes of different dimensions (Table 2). Larger particles required larger glass cuvettes to maximize the distance between the sinking particle and the wall. In general, only particles in the center of the glass cuvette were analyzed so that the distance to each wall was at its maximum. The measured sinking velocity was corrected for wall effects according to Ristow (1997)

3 $$ S_{\text{v}} = \frac{{_{\text{measured}} S_{\text{v}} }}{1 - k \times r/R} $$

where _measued_ *S*_*v*_ is the measured sinking velocity, *R* the distance between a particle and the wall, and *k* the coefficient of drag which is dependent on the shape of the cuvette. Due to the rectangular shape of the glass cuvettes with widths several times larger than depths (Table 2), only the closest walls (Z-coordinate in Fig. 1a) had the potential to noticeably influence sinking velocities. Hence, it is reasonable to assume that particles sank midway between two plain walls, in which case *k* is 1.004 (Brenner 1962). The reliability of this correction was tested by measuring sinking velocity of spherical polystyrene beads of precisely known density and comparing their measured sinking velocities with the theoretical sinking velocities calculated from Stokes’ Law (see Fig. 2). Wall effects decelerated phytoplankton sinking velocities between 1 % (*Crocosphaera watsonii*) and 7 % (*Calcidiscus leptoporus*). Small beads were slowed down by 3 % and large beads and sediment trap material by maximally 15 %.

Next to particle interactions with walls, particle interactions among each other can affect sinking velocities too. Particle–particle interactions depend (among other factors) on the distance between them, their shapes and sizes, and their orientations to each other (Happel and Brenner 1991). For example, two equally large spheres sinking directly next too each other sink roughly 30 % faster than each one would sink individually (Happel and Brenner 1991). Particle–particle interactions occur more frequently at high particle densities. It was not possible to quantify these interactions reliably within a measurement. Hence, they were avoided by keeping track of the density inside the sample and diluting the sample if necessary.

## Results

### Sinking velocities of polystyrene beads

The reliability of the method was assessed by repeated measurements of spherical polystyrene beads of known density and size and comparing the results with theoretical sinking velocities calculated from Stokes’ Law (Eq. 1). The mean sinking velocity of polystyrene beads with a certified diameter of 10 μm showed a maximum difference of 10 % based on eight repeated measurements. The difference between the mean of all eight measurements (corrected for wall effects) and the theoretical sinking velocity calculated from Stokes’ law (Eq. 1) was less then 1 % (Fig. 2a). Measured sinking velocities of beads ranging from 75 to 400 μm were increasing exponentially with increasing size as predicted by Stokes’ Law but were systematically lower than the theoretical ones due to wall effects (Fig. 2b). Correcting for wall effects with Eq. 3 improved the consistency between theoretical and measured values (Fig. 2c). The decelerating effect of the cuvette walls was growing with increasing bead diameter as predicted by Eq. 3.

### Sinking velocities of phytoplankton cells

Sinking velocities of phytoplankton cells varied with size and ballasting material such as calcium carbonate. The fastest sinking species investigated here was the calcified coccolithophore *Calcidiscus leptoporus* with about 4.3 m day^−1^, while the slowest sinking one was the un-calcified coccolithophore *Emiliania huxleyi* (0.03 m day^−1^). The presence of ballasting calcium carbonate in the coccolithophores *E. huxleyi* and *Gephyrocapsa oceanica* increased sinking velocities by about a factor of ten. Individual coccoliths of *C. leptoporus* were sinking with about 0.74 m day^−1^ which is faster than complete cells of *E. huxleyi a*nd *G. oceanica*. Phosphorus-limited *E. huxleyi* cells sank on average slightly slower than the exponentially growing ones (~0.3 and ~0.38 m day^−1^, respectively, Table 1).

The small and uncalcified cyanobacterium *Croccosphaera watsonii* with a diameter of ~3 μm had an average sinking velocity of ~0.06 m day^−1^ which is faster than the uncalcified coccolithophores *E. huxleyi* and *G. oceanica*. The cryptophyte *Rhodomonas* spec. and the silicoflagellate *Dictyocha* spec. were sinking with ~0.25 and ~0.48 m day^−1^, respectively. Note that we could not detect any signs of a silicified skeleton for *Dictyocha* spec. (Table 1).

Highest sinking velocities among the investigated diatoms were found in *Thalassiosira oceanica* (~0.2 m day^−1^), while the slowest sinking species were *Phaedactylum tricornutum* (0.065 m day^−1^). *Thalassiosira weissflogii* was by far the largest investigated diatom (ESD = 14 μm) but its sinking velocity of ~0.07 m day^−1^ was smaller than that of the other *Thalassiosira* species (Table 1).

Particle densities of spherical phytoplankton species ranged from 1,080 kg m^−3^ in *Dictyocha* spec. to 1,282 kg m^−3^ in *C. leptoporus*. Densities were higher in calcifying compared with non-calcifying species (Table 1).

### Sinking velocities of sediment trap material ranging from 80–400 μm in ESD

**Fig. 3:**
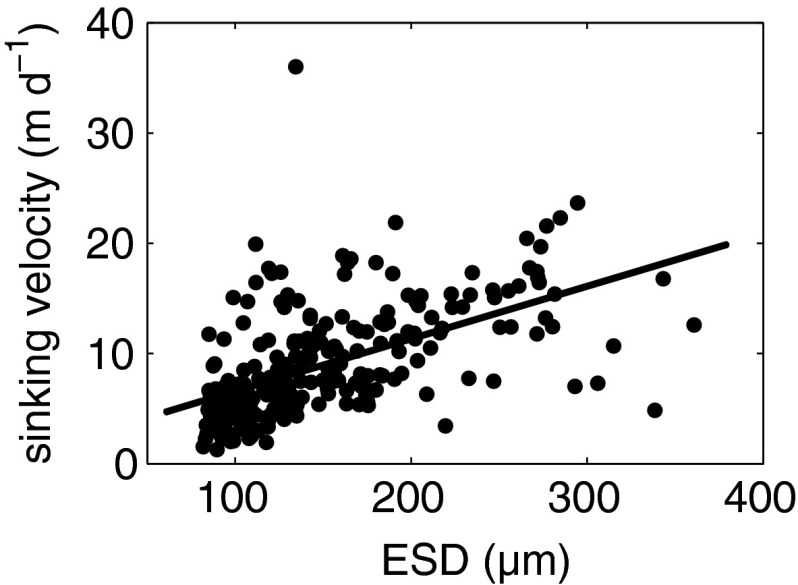
Sinking velocities of sediment trap material in relation to ESD. *R* ^2^ = 0.31, *p* < 0.0001

Sinking velocities of sediment trap particles correlated with size but showed comparatively large variations (Fig. 3). Highest sinking velocities were measured for particles that appeared relatively compact while fluffy aggregate material was sinking with the slowest rates. The average sinking velocity of all investigated particles in the size range between 80 and 400 μm was ~9 m day^−1^. Complete and fragmented fecal pellets could were sinking faster than the average sediment trap material with a mean sinking velocity of 12.5 m day^−1^ (Table 3).

**Table 3:** Average size properties and sinking velocities of all investigated fecal pellets

Sinking velocity (m day^−1^)	ESD (μm)	Length (μm)	Width (μm)	Volume × 10^6^ (μm^3^)
12.5 (±4.8)	223 (±50)	308 (±71)	95 (±22)	2.5 (±1.9)

Volume was calculated from length and width assuming cylindrical shape

### Influence of water temperature on sinking velocities

**Fig. 4:**
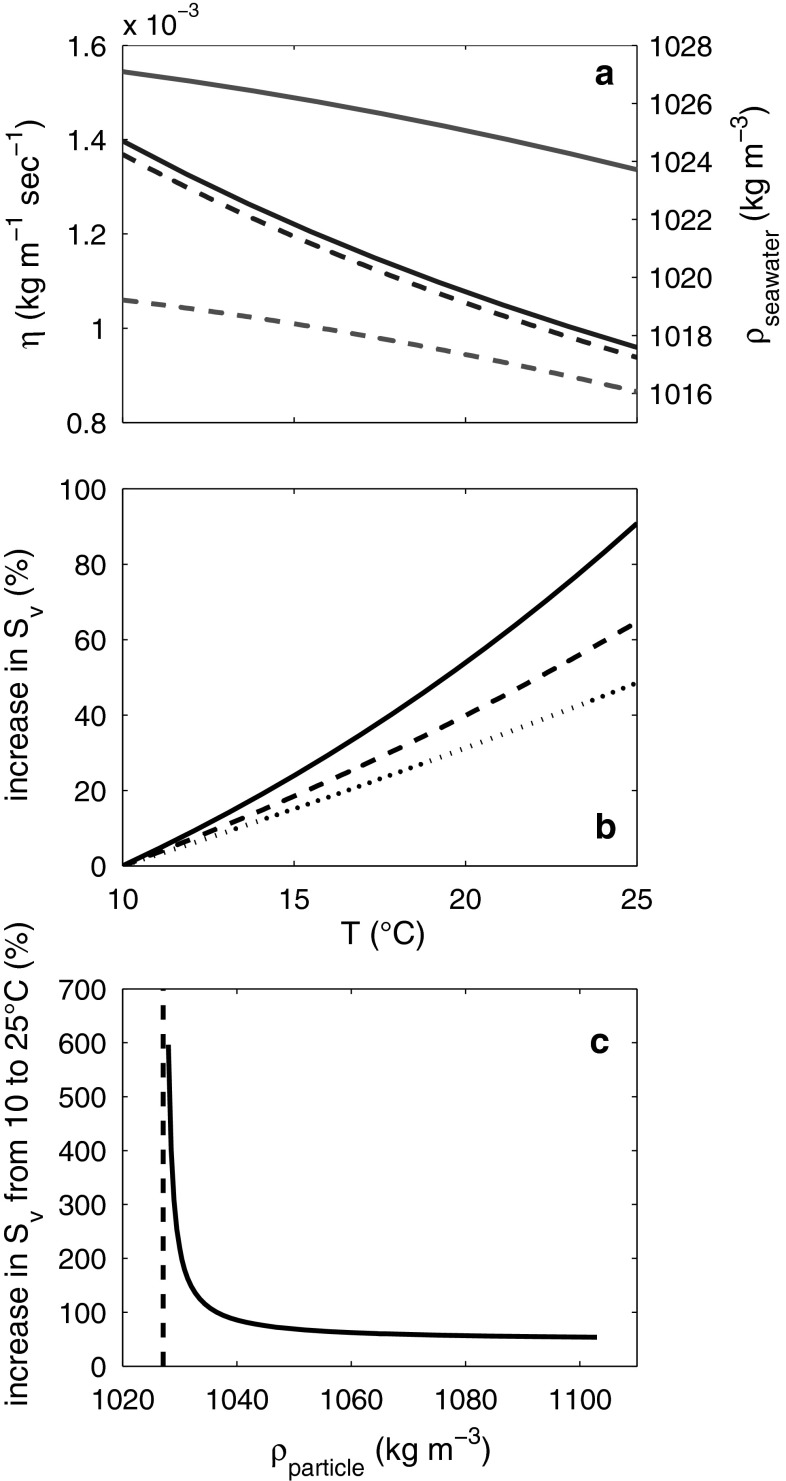
Influence of temperature on *η*_seawater_, *ρ*_seawater_, and *S*_*v*_. **a** Change of *η*_seawater_ and *ρ*_seawater_ in relation to temperature at two different salinities (*dashed lines*, *S* = 25; *straight lines*, *S* = 35). *Red lines* denote for *ρ*_seawater_ changes and *blue lines* for *η*_seawater_ changes. **b** Relative increase in *S*_*v*_ according to Stokes’ Law of particles with different density (*straight line* = *ρ*_particle_ of 1,038 kg m^−3^, *dashed line* = *ρ*_particle_ of 1,053 kg m^−3^, *dotted line* = *ρ*_particle_ of 1,200 kg m^−3^). Salinity in these calculations was 35. **c** Relative increase in particle sinking velocity from 10 to 25 °C with increasing *ρ*_particle_. The *dashed line* denotes seawater density of 1,027 kg m^−3^. Calculations in (**b**) and (**c**) were performed using Stokes’ Law

Temperature of the seawater medium had a strong influence on sinking velocities of beads ranging from 75 to 400 μm (Fig. 2a, b). To assess the relative difference of beads sinking at 10 and 19 °C, a polynomial regression was fitted through both datasets. *R* ^2^ values of these regressions were 0.98 at 10 °C and 0.97 at 19 °C, respectively. According to the fits with the polynomial regressions, beads were sinking on average 38 % faster at 19 °C than at 10 °C, which is close to the theoretical difference of 35 % calculated from Stokes’ Law. Note that the lines shown in Fig. 2b and c are not the regressions but theoretical sinking velocities calculated from Stokes’ Law. The relatively strong influence of temperature on sinking rates results from the close coupling of temperature with seawater density and viscosity (Fig. 4a). Warm water is less dense and viscous than cold water and particles sinking according to Stokes’ Law will therefore sink faster in warmer water (Fig. 2b, c; Eq. 1).

Further theoretical considerations with Stokes’ Law showed that the influence of temperature on sinking velocities is more pronounced when the density of the sinking particle gets closer to the density of the seawater medium. A particle with a density of 1,038 kg m^−3^ sank ~90 % faster at 25 °C compared with 10 °C. The sinking velocity of particles with a density of 1,200 kg m^−3^, however, increased only about 50 % over the same temperature range (Fig. 4b). The effect of temperature increased exponentially the closer the density of the sinking particle approached the density of the seawater medium (Fig. 4c).

## Discussion

### Applicability of the method

The method for sinking velocity determinations of particles between ~3–400 μm in ESD presented here is easy to set up and permits the processing of a comparatively high number of samples per day. The very good agreement of measured (FlowCAM^®^) and calculated (Stokes’ Law) sinking velocities of polystyrene beads with a representative size range indicates that our method is useful to determine sinking rates of marine (and limnic) particles and results are not biased by convection or wall effects. Nevertheless, there are some aspects of the method, which have to be discussed in more detail.

Due to the relatively short distance over which particles are tracked in the display window of the camera, it becomes difficult to measure particles sinking faster than ~200 m day^−1^. Such fast sinking particles would pass the area monitored by the camera too quick to get a reasonable amount of pictures even at the highest frame rate. The method is, therefore, inadequate to determine sinking velocities of large and heavily ballasted fecal pellets which can sink several hundreds or even thousands of meters per day (e.g. Bruland and Silver 1981; Ploug et al. 2008). Furthermore, the short distance the particles are tracked for can lead to higher variability in measured average sinking velocities in case particles are non-spherical. For example, relatively long cylindrical particles such as fecal pellets will sink faster if they are oriented vertically in the water column compared with a horizontal orientation (Holland 2010). It must, therefore, be assured to measure a relatively high number of these particles so that the number of vertically to horizontally oriented particles is balanced.

### Comparison with other methods: Phytoplankton

There is a variety of other methods available which have been successfully applied to quantify phytoplankton sinking velocities in earlier studies. Phytoplankton sinking velocities were usually measured with non-optical methods (Eppley et al. 1967; Bienfang 1981), although direct measurements using a video camera and laser scanning techniques have been established more recently (O’Brien et al. 2006; Walsby and Holland 2006). The most commonly applied method to determine sinking velocities of phytoplankton cells is the SETCOL method (Bienfang 1981). Here, sinking velocities are measured as change in bulk biomass per unit time. The difference between the SETCOL method and optical measurements with a camera is that the former monitors changes in bulk biomass distributions while individual cells are targeted in the latter. Advantages and disadvantages of the SETCOL method and direct optical measurements using a video camera are discussed in detail by O’Brien et al. (2006). They argue that video methods permit comparisons of sinking velocities with cell size, which is not possible with the SETCOL method. On the other hand, the SETCOL method could be advantageous if particles are sinking homogenously because in this case sinking velocities of a whole population and not only of a small number of particles can be investigated.

A direct comparison between sinking velocities of the coccolithophore *E. huxleyi* measured with the SETCOL method (Lecourt et al. 1996) and the FlowCAM^®^ method presented here shows that results are slightly different. Coccolith bearing and naked cells were sinking with up to ~0.5 and 0.3 m day^−1^, respectively, when measured with the SETCOL method (Lecourt et al. 1996), while they were sinking with ~0.4 and ~0.03 m day^−1^ when measured with the FlowCAM^®^ (Table 2). These differences, however, do not necessarily have to be attributed to the different measuring techniques but could rather result from different culture conditions. Bienfang et al. (1982) and Waite et al. (1997) measured sinking velocities of different diatom species with the SETCOL method. Nutrient replete cells of *Chaetoceros graciale* (length of 8.2 μm) were sinking with ~0.15 m day^−1^ (Bienfang et al. 1982), comparable with sinking velocities of similar sized cells reported here (Table 2). Furthermore, Waite et al. (1997) reported sinking velocities of ~0.07 m day^−1^ for *Thalassiosira pseudonana* and ~0.06 m day^−1^ for *Thalassiosira weissflogii* which is also in reasonable agreement with 0.13 and 0.07 m day^−1^ determined here (Table 2).

### Comparison with other methods: Sediment trap material

Sediment trap material was carefully partitioned with a 300-μm sieve prior to the measurements (see section on “Sample preparation”). During this procedure, large and robust aggregates remained on the sieve, while large and more fragile aggregates disintegrated and passed the sieve as smaller particles. The exclusive measurement of small aggregates and fragments of larger ones can lead to a systematic underestimation of the particle flux in the water column because it does not take the aggregation potential and the in situ size of the investigated material into account. Nevertheless, the method has the potential to provide valuable information on the ballasting effect of various biogenic or lithogenic materials because ballasting material influences particles of every size class.

Compared with aggregates, fecal pellets are much more resistant to mechanical stress due to the peritrophic membrane that surrounds them. Fecal pellets seemed unaffected by the sieving procedure and breakup or disintegration could not be observed during handling of the sample. Good knowledge of fecal pellet sinking velocities is important since they are, at least in some marine regions, the most important transport vehicle for organic carbon into the deep ocean (Turner 2002). In the following, we will focus on the comparison of sinking velocities of fecal pellets, since there are more data available which can be used for comparisons.

Sinking velocities of zooplankton fecal pellets range from ~5 (Paffenhöfer and Knowles 1979) up to more than 2,000 m day^−1^ (Bruland and Silver 1981) and are generally higher than the mean sinking velocity of 12.5 m day^−1^ reported here. This discrepancy is unlikely resulting from the applied method since measured sinking velocities of polystyrene beads in the same size range as fecal pellets were in good agreement with theoretical values calculated from Stokes’ Law (Fig. 2a, b).

Fecal pellet sinking velocities were shown to increase with particle size and density (reviewed in Turner 2002). Fecal pellet size cannot explain the relatively slow sinking velocities reported in our study because size and volume of the pellets (Table 3) are not fundamentally different compared to other studies where higher sinking velocities were reported (Turner 1977; Yoon et al. 2001; Ploug et al. 2008). Relatively slow sinking velocities should, therefore, rather be caused by smaller excess densities. Fecal pellet density was shown to depend on the amount of ballast material such as calcium carbonate or silicate incorporated by the pellet producers (e.g., Honjo 1976; Small et al. 1979; Harris 1994; Ploug et al. 2008). The phytoplankton biomass in the mesocosm on the days before sampling was dominated by species not forming ballasting material, with only a comparatively small fraction contributed by the small diatom *Arcocellulus* spec. This suggests low ballast loading of fecal pellets. Furthermore, the phytoplankton community was in a post-bloom phase during the time of sampling and the total biomass was low. Small et al. (1979) reported a decreased density and compactness of fecal pellets and sometimes almost empty peritrophic membranes when copepods were incubated in seawater with very low food source. The same phenomenon was also described by Yoon et al. (2001) and Bruland and Silver (1981) when zooplankton was kept for a long time in the same container which was probably depleted in food. Low ballast loading and low food availability for pellet producers could therefore explain the relatively slow sinking velocities of fecal pellets reported in this study (Table 3).

Next to fecal pellets properties, water temperature of the media at which sinking velocities are determined can make a significant difference (Figs. 2a, b, 4b, c, see the following section). Water temperature was 10 °C during measurements, which is lower than in most other studies. The same fecal pellets would sink ~70 % faster at 25 °C, assuming a fecal pellet density of 1,050 kg m^−3^.

### Effect of seawater temperature on sinking velocities

Density and viscosity of seawater are a function of temperature and salinity (Sharqawy et al. 2010). A particle with a Reynolds number smaller than ~0.5 will sink faster in less viscous and dense seawater (Fig. 4b). Hence, temperature and salinity indirectly influence sinking velocities. In the following, we will focus on the temperature component, since it strongly influences sinking velocities of particles with high and low densities, while salinity only influences sinking velocities considerably if the particle density is very close to that of the surrounding seawater.

The temperature-dependent term (*f*(*T*)) in Stokes’ Law is given by

4 $$ f (T )= \frac{{\rho_{\text{particle}} - \rho_{\text{seawater}} }}{{\eta_{\text{seawater}} }} $$

The term in the numerator is the excess density of the particle. It is very influential on *f*(*T*), and therefore, on sinking velocities, if the difference between *ρ*_particle_ and *ρ*_seawater_ is less than ~1 % (Fig. 4c). The temperature effect should therefore be most pronounced on marine aggregates that have excess densities lower than 1 % (Iversen and Ploug 2010). However, this straightforward estimate of the influence of changing *ρ*_seawater_ neglects that *ρ*_particle_ itself could possibly also change when it is transferred into warmer water. This change could result from the particular structure of aggregates. Aggregates are usually very fluffy (Iversen and Ploug 2010) and consist to a large extent of water. The density of the water inside the aggregate, and therefore, *ρ*_particle_ will also decrease when it is transferred to warmer water. Hence, the excess density of the aggregate would change differently in such a case when not only changes in *ρ*_seawater_ are accounted for. The influence of seawater temperature on excess density is, therefore, particularly hard to quantify.

In contrast, the influence of changing viscosity is easier to quantify since particle properties do not come into play. The influence of viscosity on *f*(*T*) in Eq. 4 becomes more pronounced with increasing particle excess density. Passive sinking of more compact organic matter such as bacteria (Logan and Hunt 1987) or fecal pellets (Komar et al. 1981) that do not control their buoyancy would therefore be mostly influenced by temperature driven viscosity changes. For example, a sinking particle (*Re* < 0.5; *ρ*_particle_ = 1,140 kg m^−3^; no buoyancy control) would sink ~30 % faster in the surface water of temperate regions (19 °C) than in higher latitude surface waters (10 °C). Furthermore, (assuming particle properties do not change during settling) sinking velocities would decrease when entering cooler deep water masses and retain at thermoclines where sharp temperature gradients cause an abrupt slowdown.

It is also interesting to consider the temperature effect on sinking velocities in the context of projected climate change (IPCC 2007). A marine biogeochemical model based on the rather moderate CO_2_ emission scenario SRES A2 (IPCC 2007) projects a mean surface ocean temperature increase in approximately 2 °C until 2100 AD (Schmittner et al. 2008). According to this projection, particles with sinking velocities following Stokes’ Law and an excess density between 30–180 kg m^−3^ would sink approximately 6 % faster in the surface ocean in 2100 compared with 2000 AD. Of course, it would be an extreme oversimplification to conclude that this translates in an enhanced future carbon export of the same percentage since climate change does probably affect many other parameters with strong feedback on carbon export such as nutrient distribution, primary production or bacterial remineralization (reviewed in Riebesell et al. 2009). Nevertheless, this “viscosity effect” might have the potential to slightly modify expected changes of carbon export into the deep.

## Conclusions

According to the data provided in this study, we come to the following conclusions: (1) The proposed method of measuring sinking velocities with a FlowCAM^®^ is easy to set up and provides reliable data for particles within a size range of approximately 3–400 μm and sinking velocities of up to ~200 m day^−1^. (2) Sinking velocities of phytoplankton cells were in reasonable agreement with reported literature values and were highest when the cells were ballasted with calcium carbonate. (3) The comparatively slow sinking velocities of fecal pellets stemming from sediment trap material of a mesocosm experiment are probably the result of low ballast loading and low compactness of the fecal pellets, as well as comparatively low measurement temperatures. (4) Temperature can have a large influence on sinking velocities due to the temperature-dependent change in seawater density and viscosity. This might be worth considering with respect to future carbon export.

## Abbreviations

ESD: Equivalent spherical diameter
*f*(*T*): Temperature-dependent term in Stokes’ Law
*g*: Earth’s gravitational acceleration
*k*: Coefficient of drag
_measued_*S*_*v*_: Measured sinking velocity
*η*_seawater_: Dynamic viscosity of seawater
*ρ*_particle_: Density of spherical particles
*ρ*_seawater_: Density of seawater
*S*: Salinity
*T*: Temperature
*R*: Distance between particle and wall
*r*: Radius of a sphere
*R*^2^: Coefficient of determination
*Re*: Reynolds number
*S*_v_: Sinking velocity of a particle corrected for wall effects

## References

[CR1] Armstrong RA, Lee C, Hedges JI, Honjo S, Wakeham SG (2002). A new, mechanistic model for organic carbon fluxes in the ocean based on the quantitative association of POC with ballast minerals. Deep-Sea Res Pt I.

[CR2] Bienfang PK (1981). SETCOL: a technologically simple and reliable method for measuring phytoplankton sinking rates. Can J Fish Aquat Sci.

[CR3] Bienfang PK, Harrison PJ, Quarmby LM (1982). Sinking rate response to depletion of nitrate, phosphate and silicate in four marine diatoms. Mar Biol.

[CR4] Brenner H (1962). Effect of finite boundaries on the Stokes’ resistance of an arbitrary particle. J Fluid Mech.

[CR5] Bruland KW, Silver MW (1981). Sinking rates of fecal pellets from gelatinous zooplankton (salps, pteropods, doliolids). Mar Biol.

[CR6] Clegg SL, Whitfield M (1990). Application of a generalized scavenging model to time-series ^234^Th and particle data obtained during the JGOFS North Atlantic bloom experiment. Deep-Sea Res Pt I.

[CR7] Engel A, Szlosek J, Abramson L, Liu ZF, Lee C (2009). Investigating the effect of ballasting by CaCO_3_ in *Emiliania huxleyi*: I. Formation, settling velocities and physical properties of aggregates. Deep-Sea Res Pt I.

[CR8] Eppley RW, Holmes RW, Strickland JDH (1967). Sinking rates of marine phytoplankton measured with a fluorometer. J Exp Mar Biol Ecol.

[CR100] Feinberg LR, Dam HG (1998). Effects of diet on dimensions, density and sinking rates of fecal pellets of the copepod *Acartia tonsa*. Mar Ecol Prog Ser.

[CR9] Fischer G, Karakas G (2009). Sinking rates and ballast composition of particles in the Atlantic Ocean: implications for the organic carbon fluxes to the deep ocean. Biogeosciences.

[CR10] Francois R, Honjo S, Krishfield R, Manganini S (2002). Factors controlling the flux of organic carbon to the bathypelagic zone of the ocean. Global Biogeochem Cy.

[CR11] Giddings JC, Ho J (1995). Accurate measurement of density of colloidal latex particles by sedimentation field-flew fractionation. Langmuir.

[CR12] Happel J, Brenner H (1991). Low Reynolds number hydrodynamics with special applications to particulate media.

[CR13] Harris RP (1994). Zooplankton grazing on the coccolithophore *Emiliania huxleyi* and its role in inorganic carbon flux. Mar Biol.

[CR14] Holland DP (2010). Sinking rates of phytoplankton filaments oriented at different angles: theory and physical model. J Plankton Res.

[CR15] Honjo S (1976). Coccoliths: production, transportation and sedimentation. Mar Micropaleontol.

[CR16] IPCC (2007) Climate change 2007: the physical science basis. Contribution of working group I to the fourth assessment report of the intergovernmental panel on climate change. Cambridge University Press, Cambridge

[CR17] Iversen MH, Ploug H (2010). Ballast minerals and the sinking carbon flux in the ocean: carbon-specific respiration rates and sinking velocity of marine snow aggregates. Biogeosciences.

[CR18] Klaas C, Archer DE (2002). Association of sinking organic matter with various types of mineral ballast in the deep sea: implications for the rain ratio. Global Biogeochem Cy.

[CR19] Komar PD, Morse AP, Small LF (1981). An analysis of sinking rates of natural copepod and euphausiid fecal pellets. Limnol Oceanogr.

[CR20] Lecourt M, Muggli DL, Harrison PJ (1996). Comparison of growth and sinking rates of non-coccolith- and coccolith-forming strains of *Emiliania huxleyi* (Prymnesiophyceae) grown under different irradiances and nitrogen sources. J Phycol.

[CR21] Logan BE, Hunt JR (1987). Advantages to microbes of growth in permeable aggregates in marine systems. Limnol Oceanogr.

[CR22] McNown JS, Malaika J (1950). Effect of particle shape on settling velocity at low Reynolds numbers. Trans Am Geophys Un.

[CR23] O’Brien KR, Waite AM, Alexander BL, Perry KA, Neumann LE (2006). Particle tracking in a salinity gradient: a method for measuring sinking rate of individual phytoplankton in the laboratory. Limnol Oceanogr-Meth.

[CR24] Paffenhöfer GA, Knowles SC (1979). Ecological implications of fecal pellet size, production and consumption by copepods. J Mar Res.

[CR25] Passow U (2004). Switching perspectives: do mineral fluxes determine particulate organic carbon fluxes or vice versa?. Geochem Geophy Geosy.

[CR26] Passow U, Alldredge AL, Logan BE (1994). The role of particulate carbohydrate exudates in the flocculation of diatom blooms. Deep-Sea Res Pt I.

[CR27] Pilskaln CH, Honjo S (1987). The fecal pellet fraction of biogeochemical fluxes to the deep sea. Global Biogeochem Cy.

[CR28] Ploug H, Iversen MH, Koski M, Buitenhuis ET (2008). Production, oxygen respiration rates, and sinking velocity of copepod fecal pellets: direct measurements of ballasting by opal and calcite. Limnol Oceanogr.

[CR29] Ploug H, Terbrüggen A, Kaufmann A, Wolf-Gladrow D, Passow U (2010). A novel method to measure particle sinking velocity in vitro, and its comparison to three other in vitro methods. Limnol Oceanogr Methods.

[CR30] Riebesell U, Körtzinger A, Oschlies A (2009). Sensitivities of marine carbon fluxes to ocean change. Proc Natl Acad Sci USA.

[CR31] Ristow GH (1997). Wall correction factor for sinking cylinders in fluids. Phys Rev E.

[CR32] Schmittner A, Oschlies A, Matthews HD, Galbraith ED (2008) Future changes in climate, ocean circulation, ecosystems and biogeochemical cycling simulated for a business-as-usual CO_2_ emission scenario until year 4000 AD. Global Biogeochem Cy 22:GB1013

[CR33] Sharqawy MH, Lienhard JH, Zubair SM (2010). Thermophysical properties of seawater: a review of existing correlations and data. Desalin Water Treat.

[CR34] Small LF, Fowler SW, Ünlü MY (1979). Sinking rates of natural copepod fecal pellets. Mar Biol.

[CR35] Turner JT (1977). Sinking rates of fecal pellets from the marine copepod *Pontella meadii*. Mar Biol.

[CR36] Turner JT (2002). Zooplankton fecal pellets, marine snow and sinking phytoplankton blooms. Aquat Microb Ecol.

[CR37] Uhlherr PHT, Chhabra RP (1995). Wall effect for the fall of spheres in cylindrical tubes at high Reynolds number. Can J Chem Eng.

[CR38] Waite A, Fisher A, Thompson PA, Harrison PJ (1997). Sinking rate versus cell volume relationships illuminate sinking rate control mechanisms in marine diatoms. Mar Ecol Prog Ser.

[CR39] Walsby AE, Holland DP (2006). Sinking velocities of phytoplankton measured on a stable density gradient by laser scanning. J R Soc Interface.

[CR40] Yoon WD, Kim SK, Han KN (2001). Morphology and sinking velocities of fecal pellets of copepod, molluscan, euphausiid, and salp taxa in the northeastern tropical Atlantic. Mar Biol.

